# Parallel short sequence assembly of transcriptomes

**DOI:** 10.1186/1471-2105-10-S1-S14

**Published:** 2009-01-30

**Authors:** Benjamin G Jackson, Patrick S Schnable, Srinivas Aluru

**Affiliations:** 1 Department of Electrical and Computer Engineering, Iowa State University, Ames, IA 50011, USA; 2 Center for Plant Genomics, Iowa State University, Ames, IA 50011, USA

## Abstract

**Background:**

The *de novo *assembly of genomes and transcriptomes from short sequences is a challenging problem. Because of the high coverage needed to assemble short sequences as well as the overhead of modeling the assembly problem as a graph problem, the methods for short sequence assembly are often validated using data from BACs or small sized prokaryotic genomes.

**Results:**

We present a parallel method for transcriptome assembly from large short sequence data sets. Our solution uses a rigorous graph theoretic framework and tames the computational and space complexity using parallel computers. First, we construct a distributed bidirected graph that captures overlap information. Next, we compact all chains in this graph to determine long unique contigs using undirected parallel list ranking, a problem for which we present an algorithm. Finally, we process this compacted distributed graph to resolve unique regions that are separated by repeats, exploiting the naturally occurring coverage variations arising from differential expression.

**Conclusion:**

We demonstrate the validity of our method using a synthetic high coverage data set generated from the predicted coding regions of *Zea mays*. We assemble 925 million sequences consisting of 40 billion nucleotides in a few minutes on a 1024 processor Blue Gene/L. Our method is the first fully distributed method for assembling a non-hierarchical short sequence data set and can scale to large problem sizes.

## Background

### Introduction

The development of high-throughput short sequencing technologies, such as the Illumina Solexa and Applied Biosystems Solid systems, has sparked renewed interest in sequence assembly. The promise of inexpensive short reads has opened the door to the possibilities of resequencing individuals and sequencing more organisms at lower cost.

An important problem in short sequence assembly is *de novo *genome reconstruction. For genomes with high repeat content, this task is already difficult with the much longer Sanger reads [1]. For accurate assembly of short sequences, many have proposed using more rigorous graph models rather than to the overlap-based greedy heuristics often utilized for Sanger reads. Graph models of particular interest include De Bruijn graphs and string graphs in either directed or bidirected forms.

As graph models of assembly are compute and memory intensive, and the coverage needed with short read technologies is large, it is difficult to validate the proposed methods on large eukaryotic genomes. Pevzner *et al. *[2] originally tested the EULER assembler using bacterial genomes. Myers [3], Medvedev *et al. *[4], and Hernadez *et al. *[5] also demonstrate their methods on prokaryotes. Zerbino *et al. *[6], Warren *et al. *[7] and Dohm *et al. *[8] validated their methods using single BACs.

Butler *et al. *[9] computed an assembly of 39 million bases in 2 days using a database and a workstation with 64 gigabytes of RAM. They use a modified directed string graph model for assembly, and require clone pairs of three different lengths to achieve the result. Their paper, while presenting a sequential method, does demonstrate that the *de novo *assembly of long genomes using very short shotgun sequences is possible.

Sundquist *et al. *[10] propose the SHRAP hierarchical short sequence protocol and method for assembling hierarchical data in parallel. The hierarchical nature of their problem results in a natural decomposition into smaller problems that can be distributed, which is fundamentally different from the problem of assembling shotgun data in parallel, which we present here.

In this paper, we present a method for assembling the transcriptome of an organism from short reads derived from unnormalized expression libraries. We follow Myers' and Medvedev's lead [3,11] and model the assembly problem as that of finding a tour of a bidirected string graph, which we consider a natural model. Importantly, we address the challenges of constructing and manipulating this graph using multiprocessor computers. In addition to speeding up the assembly process, the main benefit of using such machines is the large amount of memory available for the manipulation of the graph for large problems.

Our method is a fully distributed parallel method that can process high coverage data sets and quickly reconstruct the underlying sequences. First, we construct the distributed bidirected string graph. Once the graph has been constructed, we identify and compact chains within the graph, which correspond to unique long contigs. The final step of the algorithm is to process the graph in such a way that we can reduce the edges, and, correspondingly, increase the length of each edge, or the length of each contig in the assembly. In this manipulation, we make novel use of the variation in sequence coverage of the transcriptome naturally arising due to differential expression. Coverage has been used in assembly methods before, particularly in transforming the assembly problem to that of network flow [3]. However, instead of using uniform coverage as do these methods, our method leverages non uniform coverage.

We analyze the error in Solexa data and then use this analysis to generate synthetic data for the maize (*Zea mays*) transcriptome. We then use a parallel implementation of our method to assemble 40 billion bases in a few minutes on a 1024 node Blue Gene/L computer. We validate the method by aligning our assembled contigs back to the reference genome.

### Model of parallel computation

To ensure practical applicability, we use the distributed memory model of parallel computation. Each processor has access to its local memory, and remote memory access is achieved through communication over an interconnection network. The run-time of an algorithm is characterized by the parallel computation time and communication time. We use the permutation network model, in which each processor can simultaneously send/receive a message of *m *bytes provided no two source/destination processors have the same id. The communication complexity is then measured by the number of such communication rounds, and the total volume of parallel communication. The former accounts for the number of times the expensive latency cost is paid, while the latter accounts for the cost of network routing.

Let *p *denote the number of processors. We make use of the regular all-to-all communication primitive, in which each processor sends a distinct message of *O*($\frac{n}{p^{2}}$) bytes to every other processor (i.e., one communication round with *O*($\frac{n}{p}$) parallel communication volume). A many-to-many communication is similar, except that each processor sends and receives variable sized chunks of data. A bounded many-to-many communication can be made to behave as a regular all-to-all communication with total size *r *+ *s*, where *s *is the total number of elements sent by any processor and *r *is the maximum number of elements received by any processor [12].

When we refer to an element in an array sending a message to another element in an array, we implicitly mean that each processor will collect all such messages and send and receive them using a many-to-many communication before routing them to their final array destination.

Parallel sorting is an important subroutine in our method, and the best algorithm for parallel sort on distributed memory machines that achieves a good final distribution of the sorted values is regular sample sort [13]. A regular sample sort uses a constant number of bounded many-to-many communications and *O *($\frac{n}{p}$) local computation for integer sort, and *O *($\frac{n}{p}\log\frac{n}{p}$) local computation time for comparison sort.

A primary concern in the development of parallel algorithms is to demonstrate that the algorithm scales well as the number of processors increases. This allows one to handle larger problem sizes by using larger machines without compromising on time to solution. Perfect speedup is characterized as linear speedup with number of processors.

### The bidirected graph model

In a bidirected graph *G *= {*V*,*E*}, each edge has two directions, one associated with each incident node. For each ordered pair of nodes (*u*,*v*) there are four possible connecting edges: *u*▷-▷*v*, *u*◁-◁*v*, *u*◁-▷*v*, and *u*▷-▷*v*. Edges are represented by tuples <*u*, *v*, *d*_*u*_, *d*_*v*_>, with *d*_*u*_, *d*_*v *_∈ {▷, ◁}. For each unordered pair of nodes {*u*, *v*} exactly two such tuples exist, one for each of the ordered pairs (*u*, *v*) and (*v*, *u*), respectively. Accordingly, we represent the bidirected graph as a distributed tuple list, two tuples per edge.

In this representation, sorting tuples by node labels will distribute edges such that all edges adjacent to a given node reside in the same processor. Alternatively, sorting tuples by a canonical representation (for example considering the smaller node ID followed by the larger node ID) will move both tuples corresponding to an edge to the same processor.

**Figure 1:**
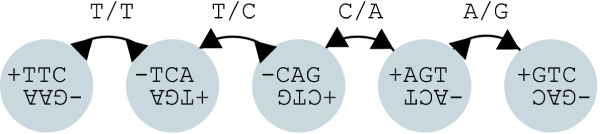
**Bidirected graph**. The bidirected model for use in assembly. The figure shows the four edge types as described in the text, as well as the corresponding edge labels in the string graph.

The sequence assembly problem is naturally modeled as a bidirected graph (See Fig. 1) [3,11]. Consider each input sequence as a DNA molecule by taking both the sequence and its complementary strand. By convention, we label the lexicographically larger of the two strands as '+', and the lexicographically smaller of the two strands as '-'. We begin with a bidirected De Bruijn graph of the input sequences [11] and transform it into a bidirected string graph, which is an edge labeled graph upon which some traversal of the graph corresponds to the underlying genomic sequence [3].

In the bidirected De Bruijn graph, each node *u *corresponds to a *k*-molecule present in some input sequence. We label its two strands by *u*^+ ^and *u*^-^. If two such molecules *u *and *v *contain a *k *- 1 length overlap, they can do so in four possible ways, each of which directly corresponds to the types of edges in a bidirected graph.

• Case I: The (*k *- 1)-length suffix of *u*^+ ^is a prefix of *v*^+^. This is denoted *u*▷-▷*v*.

• Case II: The (*k *- 1)-length suffix of *u*^- ^is a prefix of *v*^-^. This is denoted *u*◁-◁*v*.

• Case III: The (*k *- 1)-length suffix of *u*^- ^is a prefix of *v*^+^. This is denoted *u*◁-▷*v*.

• Case IV: The (*k *- 1)-length suffix of *u*^+ ^is a prefix of *v*^-^. This is denoted *u*▷-◁*v*.

The bidirected De Bruijn graph can be easily converted into a bidirected string graph, with two character labels on each edge, *c*_*u *_and *c*_*v *_where *c*_*u *_corresponds to the next character on the DNA molecule when traveling away from *u *along the edge, and *c*_*v *_corresponds to the next character on the DNA molecule when traveling away from *v *along the edge. This data is added to the edge tuple, resulting in tuples of the form ⟨*u*, *v*, *d*_*u*_, *d*_*v*_, *c*_*u*_, *c*_*v*_⟩.

A valid path in a bidirected graph is any ordered sequence of tuples ⟨*e*_1_, *e*_2_, ... *e*_*x*_⟩, where *e*_*i *_= ⟨*u*_*i*_, *v*_*i*_, $d_{u_{i}}$, $d_{v_{i}}$⟩, such that *v*_*i *_= *u*_*i*+1 _and $d_{v_{i}}\neq d_{u_{i+1}}$ for all consecutive tuples *e*_*i *_and *e*_*i*+1 _in the path.

Conceptually, what it means to travel along an edge that "changes direction" is to align the positive strand in node *u *to the negative strand in node *v*. To travel along an edge that maintains its direction is to align the positive to positive. One advantage of this model is that a single tour of the graph is used to construct both strands of the double stranded DNA simultaneously. Another advantage is that the number of nodes in the graph is reduced by half when compared to a directed graph model.

## Methods

### Parallel graph construction

We are given *m *sequences of total length *n*, sampled from a genome of total length *g*, distributed among *p *processors such that there are $\frac{n}{p}$ bases per processor. We wish to construct a bidirected string graph with *O*(*g*) edges and nodes, distributed among processors such that each processor knows all edges adjacent to *O *($\frac{g}{p}$) nodes. For a detailed description and analysis of graph construction, as well as refinements to the basic algorithm presented here, see Jackson *et al. *[14].

We represent each *k*-molecule in the input sequence as a base 4 number (in 2*k *bits) using its lexicographically larger stand. These representatives can then be sorted in parallel, with identical elements merged into one. Due to the 4-letter DNA alphabet, a *k*-molecule *u *could overlap with at most 8 *k*-molecules *v*. We construct the messages to be sent to hypothetical molecules *v *that could be attached to *u*, such that for all such molecules, either *v *will send a message to *u *or *u *will send a message to *v*.

The messages are constructed for each of the three ways in which *u *can overlap with *v*. We construct each message such that it can be sent to the representative of *v *(we target the hypothetical positive strand). For each message, we construct a tuple ⟨*id*, *dest*, *type*, *char*⟩, where *id *is the node id of *u*, *dest *is the representative of *v*, *type *is the type of the message, which will inform the type of edge to draw in the graph, and *char *is the character to be associated with the edge when moving from *u *to *v*. Each hypothetical edge in the bidirected De Bruijn graph is thus represented by exactly one message.

For each message ⟨*id*, *dest*, *type*, *char*⟩ received by *k*-molecule *v*, we generate two tuples. The resulting tuple list is sorted and any duplicates are removed, resulting in a distributed tuple list representation of the graph.

Using a linear time radix sort, parallel graph construction is achieved in *O *($\frac{n}{p}$) parallel compute time, *O*(1) communication rounds, and *O *($\frac{n}{p}$) parallel communication volume.

#### Dealing with error

Pevzner et al. [2] and Dohm et al. [8] deal with erroneous sequences by editing those that have suspicious *k*-mers. The idea is that, given high coverage, errors will manifest themselves in the sequences as *k*-mers that occur only once. This is because as long as error is not systematic, the likelihood of seeing the same error twice at the same position is low. If a sequence containing a suspicious *k*-mer can be uniquely edited into a valid sequence, then the editing is done; if not, the sequence is discarded.

This approach, being a preprocessing step, can be used in conjunction with any assembly method to greatly reduce error in input sequences, and many recent works on assembly have advocated its use. We can use the same concept to identify error at a later stage in the method, by removing the offending *k*-mers from the bidirected De Bruijn graph.

### Parallel identification of unique contigs

The bidirected graph generated in the previous section will likely have many long chains, each corresponding to sequences that can be unambiguously assembled into a single contig. These chains are then connected in a more interesting topology that must be further analyzed. Will will compact these chains (forming a single edge in the graph for each chain) using undirected list ranking.

#### Weighted undirected list ranking problem

For the undirected list ranking problem, we are given a set of weighted, undirected lists of total length *n *as an array of tuples ℒ[*u*] = ⟨*A*_1_, *W*_1_, *A*_2_, *W*_2_⟩ of size *n*, where *u.A*_1 _and *u.A*_2 _hold pointers to the two nodes adjacent to node *u*, and *u.W*_1 _and *u.W*_2 _hold the corresponding weights. If *u *is an endpoint, then either *u.A*_1 _or *u.A*_2 _will point to *u*. If *u *is the sole element of a list, then both *u.A*_1 _and *u.A*_2 _will point to *u*. If *u.A*_*i *_= *v*, then either *v.A*_1 _= *u *or *v.A*_2 _= *u*. If *u.A*_*i *_= *v *and *v.A*_*j *_= *u*, then *u.W*_*i *_= *v.W*_*j*_.

For the undirected list ranking problem, we wish to compute the tuple R[*u*] = ⟨*R*_1_, *E*_1_, *R*_2_, *E*_2_⟩. *u.R*_1 _is the rank of *u *relative to *u.E*_1_, the list endpoint in the direction of *u.A*_1_. *u.R*_2 _and *u.E*_2 _are respectively defined in the direction of *u.A*_2_.

#### List ranking transformation

Conceptually, graph compaction involves replacing all chains in the graph with single edges, labeled by the concatenation of all edge labels along the chain. We will now show how to transform this problem to the problem of undirected list ranking. Consider edge tuples ⟨*u*, *v*, *d*_*u*_, *d*_*v*_, *c*_*u*_, *c*_*v*_⟩ augmented with two additional pieces of information *id *and *adj*. We will transform the graph compaction problem to the undirected list ranking problem using the following algorithm:

1. Sort all tuples with the smaller node id as the primary key and the larger node id as the secondary key. This results in both tuples for a given edge coming together in the sorted order.

2. If necessary, shift boundary tuples to guarantee that no edge is split between processors.

3. Give each pair of tuples a unique ID in the range 1 to |*E*|.

4. Sort all tuples with the first node id as the primary key and the second node id as the secondary key. This results in all tuples for a given node coming together in the sorted order.

5. If necessary, shift boundary tuples to guarantee that all tuples with the same first node id are on the same processor.

6. For each set of tuples $A_{u}$ sharing the first node id *u*:

(a) If $A_{u}$ = {*x*, *y*} and *x.d*_*u *_≠ *y.d*_*u *_(there is a valid path through this node in the graph), then set *x.adj *← *y.ID *and *y.adj *← *x.ID*.

(b) Otherwise, for all tuples *x *∈ $A_{u}$ set *x.adj *← *x.id*.

7. Sort all tuples with the smaller node id as the primary key and the larger node id as the secondary key. Shift boundary tuples as necessary.

8. For each pair of tuples *x *and *y *corresponding to the same edge, set ℒ[*id*] ← ⟨*x.adj*, 1, *y.adj*, 1⟩.

The runtime of the transformation is dominated by a constant number of parallel sort operations.

#### Parallel list ranking

The undirected list ranking problem is a modification of the traditional list ranking problem, which has been extensively studied on parallel computers. The sparse ruling set algorithm achieves the best run time on large data sets with a large number of processors [15], and we have accordingly designed a modified version of the sparse ruling set algorithm for undirected lists.

The sparse ruling set algorithm is a recursive algorithm on a weighted list (each edge is associated with a weight or distance). In the base case, the lists are gathered to one processor and solved using a serial list ranking algorithm, in linear time.

For the inductive case of the algorithm, we wish to achieve the following objectives. First, we wish to mark some subset of nodes which include all endpoints and some other nodes. Second, we wish to find the distance between each unmarked node and its two closest marked nodes. Finally, we wish to find the distance between adjacent marked nodes.

**Figure 2:**
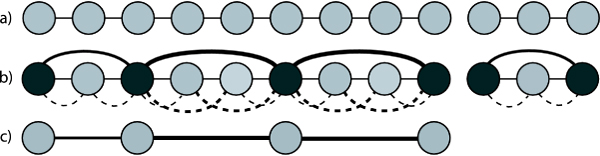
**Sparse ruling set algorithm**. The recursive step of the sparse ruling set algorithm. a) The input lists. b) The marked list with edges drawn between marked nodes, as well as pointers from unmarked nodes to nearest marked nodes (dashed). Edge weights are shown via line widths. c) The recursive problem (notice that list on the right has been completed and does not form a recursive subproblem).

Once we have this information, we will set the adjacencies of each marked node to the nearest marked nodes in the list, and the weights as the distance to those marked nodes, and recursively solve the problem (see Fig. 2). After the recursion, we will know R for all marked nodes. We can use the stored distance information from the unmarked nodes to the marked nodes to compute R for all unmarked nodes.

We will now formally describe an in place recursive algorithm. The algorithm communicates messages with four components: ℳ = ⟨*t*, *s*, *m*, *r*⟩, where *t *is the target of the message, *m *is the id of the originating marked node, *s *is the source of the message, and *r *is the distance to the originating marked node.

For each node *u *we define *u.i*, an integer marking of the node. Let *l *identify the level of recursion. *u.i *and *l *will be used in conjunction to identify unmarked nodes and marked nodes for each recursion level, allowing for an in place algorithm. For each level *l*, *u.i *= *l *if and only if then *u *is an unmarked node. *u.i *= *l *+ 1 if and only if *u *is a marked node. Initially *u.i *= 0 for all *u*. Initially *l *= 0. Let *n*_*l *_be the number of nodes with *u.i *= *l*. We execute the following recursive algorithm:

1. **Mark nodes: **For each node *u *that is unmarked (*u.i *= *l*), mark *u *(*u.i *← *l *+ 1) under the following conditions:

• *u *is an end point.

• with some probability *ρ*.

2. **Construct messages: **For each node *u *that is a marked node, construct messages to be sent to the neighbors of *u*: ⟨*u*.*A*_1_, *u*, *u*, *u*.*W*_1_⟩ and ⟨*u*.*A*_2_, *u*, *u*, *u*.*W*_2_⟩

3. **Propagate messages: **While there exist some messages to send:

(a) Send and receive all messages. This is a many-to-many communication.

(b) For each message ℳ received with target *t*, we can get the origin of the message by comparing *s *with *t.A*_1 _and *t.A*_2_. We will assume that *s *= *t.A*_1_; the other case is handled similarly.

• If *t *is a marked node then set the new adjacencies and weights for the recursive problem: *t.A*_1 _← *m *and *t.W*_1 _← *r*.

• If *t *is an unmarked node then:

○ Record the originating marked node and the distance to it: *t.E*_1 _← *m *and *t.R*_1 _← *r*.

○ Propogate ℳ as ⟨*t*.*A*_2_, *m*, *t*, *r *+ *t*.*W*_2_⟩.

4. **Recursion: **At this point, the recursive problem has been initialized. If *n*_*l*+1 _<*T *proceed with the base case. Otherwise recurse with *l *← *l *+ 1.

5. **Recursive Result: **When the recursion is complete, all marked nodes will have R[*u*] computed.

6. **Compute **R[*u*] **for all unmarked nodes: **For each *u *with *u.i *= *l*:

(a) **Get flanking nodes**: For flanking nodes: *v *← *u.E*_1 _and *w *← *u.E*_2_, gather R[*v*] and R[*w*] if *v *and *w *are not local. Notice that *v *and *w *are marked.

(b) **Calculate **R[*u*]: It must be the case that either *v.E*_1 _= *w.E*_1 _or *v.E*_1 _= *w.E*_2_. We will consider the first case, as the second case is handled similarly.

• If (*v.R*_1 _*< w.R*_1_) then set R[*u*] ← ⟨*u.R*_1 _+ *v.R*_1_,*v.E*_1_,*w.R*_2 _- *u.R*_2_,*v.E*_2_⟩.

• If (*v.R*_1 _*> w.R*_1_) then set R[*u*] ← ⟨*v.R*_1 _- *u.R*_1_,*v.E*_2_,*w.R*_2 _+ *u.R*_2_,*v.E*_1_⟩.

The base case of the algorithm requires gathering all remaining *n' *marked nodes to a single processor to be ranked. To do so, we must map the pointers in the original array of size *n *to the new array of size *n'*. We construct an additional array that maps from the domain of *n' *to the domain of *n*. Once this array is gathered to a single processor, an inverted mapping is created. This inverted mapping is used to map the adjacency pointers, which index into the global domain, to the smaller domain.

#### Run-time analysis

The number of rounds of message passing in Step 3 is given by the longest distance between two marked nodes. As each node is randomly marked, this distance is bounded by 3*p *ln(*n*_*l*_) with high probability [16]. Therefore, the expected number of communication rounds is *O*(log(*n*_*l*_)). The communication volume over these *O*(log(*n*_*l*_)) rounds is *O *($\frac{n_{l}}{p}$). Because *n*_*l *_is expected to exponentially decrease in *O*(log *n*) recursive calls, the the total expected run-time of undirected list ranking is given by *O *($\frac{n}{p}$) parallel compute time, *O*(log^2 ^*n*) communication rounds, and *O *($\frac{n}{p}$) parallel communication volume.

#### Compacted graph construction

After solving the list ranking transformation, we will set *id *and *adj *for tuples *x *and *y *as follows:

• if R[*id*].*E*_1 _≤ R[*id*].*E*_2_:

○ *x.id ← y.id ← *R[*id*].*E*_1_

○ *x.adj ← y.adj ← *R[*id*].*R*_1_

• if R[*id*].*E*_1 _> R[*id*].*E*_2_:

○ *x.id ← y.id ← *R[*id*].*E*_2_

○ *x.adj ← y.adj ← *R[*id*].*R*_2_

The *id *component of each edge tuple corresponds to the chain *id*, and the *adj *component of each edge tuple corresponds to the chain position. By sorting the tuples using these two fields as the primary key and the secondary key respectively, we can order all tuples according to their chain membership and position. If we shift boundary elements such that all elements with the same *id *are on the same processor, all tuples belonging to the same chain will be local to a processor. From these sorted tuples, we will construct our compacted graph representation.

First, we must store chains, each chain consisting of a sequence of bases. Each base in the chain is represented by a tuple ⟨*b*_1_, *b*_2_, *id*, *pos*⟩, where *b*_1_,*b*_2 _∈ {*A*, *C*,*G*,*T*}. This representation arises naturally from the tuples in the sorted order described above, and in fact the transformation to this representation only removes redundant and unnecessary information.

In addition to the chains, we also construct a distributed tuple list that models the compacted string graph. Each tuple is of the form ⟨*u*, *v*, *d*_*u*_, *d*_*v*_, *cov*, *ch*_*id*, *ch*_*dir*⟩, with *u *the first endpoint, *v *the second endpoint, *d*_*u *_the direction of the arrowhead at *u*, *d*_*v *_the direction of the arrowhead at *v*, *cov *the average coverage on that edge, *ch_id *the identifier of the chain that labels this edge, and *ch_dir *= {*forward*, *reverse*} corresponding to which strand of the chain should be read when moving from *u *to *v*.

The tuples for the compacted graph can be easily constructed by scanning the original graph tuples in the sorted order described above. For every chain starting with tuple ⟨*u*, *v*, *d*_*u*_, *d*_*v*_, *c*_*u*_, *c*_*v*_, *id*, 0⟩ and ending with tuple ⟨*x*, *y*, *d*_*x*_, *d*_*y*_, *c*_*x*_, *c*_*y*_, *id, adj*⟩, we construct tuples ⟨*u*, *y*, *d*_*u*_, *d*_*y*_, *cov*, *id*, *forward*⟩ and ⟨*y*, *u*, *d*_*y*_, *d*_*u*_, *cov*, *id*, *reverse*⟩. Assume that the coverage information for each can be calcualted as the average coverage of all positions along the chain.

### Graph reduction

At this point of graph processing, much of the repeat structure of the genome will be hidden in the graph, and as a result the length of all chains will be less than *g*. We wish to perform a sequence of reductions that will simultaneously simplify the graph while expanding the length of all chains to approach the size of *g*. We do this by performing graph manipulations centered at some nodes.

Consider the set of tuples $A_{u}$ = {*t*_1_, *t*_2_, ... *t*_*k*_} all sharing the first node id *u*. These tuples correspond to edges incident to *u *in the graph. We can partition $A_{u}$ into two sets $I_{u}$ and $O_{u}$, where *t*_*i *_∈ $I_{u}$ if and only if *t*_*i*_.*d*_*u *_= ◁, while *t*_*j *_∈ $O_{u}$ if and only if *t*_*j*_.*d*_*u *_= ▷. Thus, conceptually when traversing the graph, if we enter the node *u *along an edge that corresponds to a tuple in $I_{u}$, we must exit the node in an edge that corresponds to a tuple in $O_{u}$, and vice versa. This means that for each *t*_*i *_∈ $I_{u}$ there are |$O_{u}$| possible continuations, and for each *t*_*j *_∈ $O_{u}$ there are |$I_{u}$| possible continuations. Our goal is to reduce these possibilities.

First, we will choose some ${I^{\prime }}_{u}\subseteq I_{u}$ to remove from $I_{u}$. Next, for each *t*_*i *_∈ ${I^{\prime }}_{u}$, we define a subset ${O^{\prime }}_{u}\subseteq O_{u}$. These are the nodes from $O_{u}$ that we wish to remain connected to *t*_*i*_. We then can define ${O^{\prime }}_{u}=\cup_{i}O_{u}^{i}$ as the set of edges to remove from $O_{u}$.

When we remove graph edges corresponding to ${I^{\prime }}_{u}$ and ${O^{\prime }}_{u}$, we will replace them with the following edges. For each *t*_*i *_= ⟨*u*, *v*, *d*_*u*_, *d*_*v*_, ...⟩ ∈ ${I^{\prime }}_{u}$ and *t*_*j *_= ⟨*u*, *w*, *d*_*u*_, *d*_*w*_, ...⟩ ∈ $O_{u}^{i}$, construct new edge with tuples ⟨*v*, *w*, *d*_*v*_, *d*_*w*_, ...⟩ and ⟨*w*, *v*, *d*_*w*_, *d*_*v*_, ...⟩. We will also update the chain associated with these new edges to be the concatenation of the corresponding chains for the deleted edges. We call this sequence of operations a graph reduction centered on node *u*.

The actual choice of ${I^{\prime }}_{u}$ and ${O^{\prime }}_{u}$ result from the following rules:

#### Rule 1: Y to V reduction

**Figure 3:**
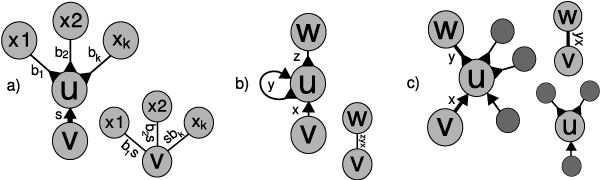
**Graph reduction rules**. The three graph reduction rules as described in the text: a) Y to V reduction b) loop reduction c) coverage matching. Each figure is labeled with a node identifier and chain identifier, and shows the structure of the graph before and after the reduction. It also shows how the underlying chains are concatenated for each type of reduction.

We show an example of this rule being applied in Fig. 3.a. A Y-node is a node in which |$I_{u}$| = 1 and |$O_{u}$| > 1 (or vice versa). We will consider the case where |$I_{u}$| = 1 and |$O_{u}$| = *k*. For the Y to V transformation, we set ${I^{\prime }}_{u}\leftarrow I_{u}$ and ${O^{\prime }}_{u}\leftarrow O_{u}^{i}\leftarrow O_{u}$. Because the adjacency list for *u *is now empty, we consider *u *removed from the graph. In essence, this rule allows for repeated elements from the genome to be duplicated in the graph. With each such operation, we would expect the total length of all edge labels in the graph to approach the actual length of the genome, but still be bounded by said length.

#### Rule 2: Loop reduction

We show an example of this rule being applied in Fig. 3.b. A loop node is a node in which $I_{u}$ = {⟨*u*, *v*, ◁, *d*_*v*_, ...⟩, ⟨<*u*, *u*, ◁, ▷, ...⟩} and $O_{u}$ = {⟨*u*, *w*, ▷, *d*_*w*_, ...⟩, ⟨*u*, *u*, ▷, ◁, ...⟩. There exists exactly one valid traversal of the graph at a loop node *u*: enter *u*, take the loop, and then exit *u*. As in the previous rule, we remove all edges adjacent to *u*, but this time we replace these adjacencies with a single edge. As shown in Fig. 3.b, the resulting chain is the concatenation of three chains (labeled *x*, *y*, and *z *in the figure).

These two rules were also described by Medvedev *et al. *[11]. Their iterative application to the graph results in a graph that Medvedev termed the *conflict graph*, consisting entirely of nodes that fall under two classes-either |$A_{u}$| = 1 or |$I_{u}$| > 1 and |$O_{u}$| > 1.

#### Rule 3: Coverage matching

We show an example of this rule being applied in Fig. 3.c. We will make use of the special nature of transcriptome data to match incoming tuples with outgoing tuples. Consider an incoming tuple *t*_*i *_and outgoing tuple *t*_*j*_. If |*cov*_*i *_- *cov*_*j*_| <*T*, where *T *is some threshold, then we term *t*_*i *_and *t*_*j *_*compatible*. If *t*_*i *_is only compatible with *t*_*j *_and *t*_*j *_is only compatible with *t*_*i*_, then we term them *uniquely compatible*. We now define ${I^{\prime }}_{u}$ to be that set of all tuples *t*_*i *_in $I_{u}$ that have a uniquely compatible tuple *t*_*j *_in $O_{u}$ and define $O_{u}^{i}$ = {*t*_*j*_}.

We have introduced this rule for the specific problem of transcriptome assembly. Through this rule we leverage the coverage information inherent in the graph to reduce the number of possible traversals of the graph.

#### Parallel graph reduction

We wish to perform the described graph reduction in parallel. In general, we will proceed in a series of iterations. In each iteration we will identify nodes that center reductions and carry out those reductions in parallel.

The first step is to find nodes that will center reductions. We can identify all nodes obeying one or more of our reduction rules in parallel because our rules require only local adjacency information, which is available on a single processor if we sort tuples by the first node ID. However, we cannot concurrently carry out reductions on all of these nodes, because if nodes *u *and *v *both center reductions, and *u *and *v *are adjacent in the graph, the operations they wish to perform will be incompatible. This is because node *u *might want to remove itself from the graph, while node *v *might wish to make a new edge with *u *as an endpoint.

For this reason during each iteration we can only operate on an independent set of the nodes identified as centering valid reductions. An independent set of nodes is a set of nodes such that the induced graph has an empty edge set. Finding a maximum independent set is NP-hard [17] (it is equivalent to finding the maximum sized clique in the complement graph). A randomized parallel algorithm for fining a *maximal *independent exists [18], but it uses *O*(log *n*) communication rounds. Instead, we describe a heuristic algorithm that chooses a large independent set of nodes assuming that the nodes have similar degree and the node identifiers are randomly permuted. When the following algorithm completes, black nodes mark an independent set.

1. Mark all nodes white.

2. For each node *u *identified as centering a reduction:

(a) Mark *u *black.

(b) Send messages to all nodes adjacent to *u*.

(c) For each black node *v *adjacent to *u*, if *u.id *> *v.id*, mark *u *white.

The second step is to carry out the reductions in parallel. For this we define a sufficient set of four operations. For each of the operations, the processor holding the reduction node sends messages to the processors holding the tuples and chains to be modified.

1. **Delete**(*u*,*v)*: Deletes two tuples.

2. **Insert**(*u*, *v*, *d*_*u*_, *d*_*v*_, *cov*, *ch_id*, *ch_dir)*: Creates two tuples for the new edge.

3. **Update***(ch_id*_*old*_, *ch_id*, *of f set*, *flip)*: Updates chain identified by *ch_id*_*old*_: sets the identifier to *ch_id*, adds *of f set *to the molecule positions, and possibly flips the orientation of the chain by reversing the order.

4. **Duplicate***(ch_id*_*old*_, *ch_id*, *of f set, flip)*: Copies the chain and then updates it.

We will now describe the parallel algorithm for graph reduction.

1. Find all reduction nodes in the graph.

2. Find an independent set of such nodes using the heuristic described above.

3. For all nodes in the independent set, create messages for updating the graph, and distribute these messages using a many to many communication.

4. Process in parallel the graph manipulation messages. This can be done using a single scan of the distributed tuple array.

5. Process in parallel the chain manipulation messages. This can be done using two scans of distributed tuple array.

6. Re-sort the graph and chain tuples to maintain sorted order.

7. If some reduction in the graph has occurred, continue with Step 1.

#### Run-time analysis

Steps 1, 2, 3, and 4 take *O *($\frac{n}{p}$) local computation, where *n *is the number of nodes in the graph, and a constant number of communication rounds. Steps 5 and 6 take *O *($\frac{g}{p}$) local computation and a constant number of communication rounds with *O *($\frac{g}{p}$) communication volume, where *g *is the size of genome. Because in practice the size the graph is much less than the size of the genome the running time of each iteration is *O *($\frac{g}{p}$), the communication volume is *O *($\frac{g}{p}$), and the number of communication rounds is *O*(1).

Because the time taken for Step 6 dominates the runtime and is independent of the number of chains being processed, we see benefit in trying to limit the number of iterations. Still, we use a heuristic rule to find an independent set of reduction nodes that works well in practice, and empirically we observe the number of iterations to be on the order of *log*(*n*). More importantly, the resulting program was able to process large inputs in a matter of seconds using this rule. Whether using the parallel randomized algorithm [18] to find a maximal independent set significantly reduces the number of iterations and improves the runtime is an open question.

### Writing the contigs

Once we have constructed the graph, compacted the chains, and finished graph reduction, we can output the contigs by traversing the final chains. The starting point for traversal will dictate which of the two strands of DNA will be written. From each chain in the graph, we can output a strand of DNA with (*l *+ *k *- 1) nucleotides, where *l *is the length of the chain (See Fig. 1). This is because the strand of DNA read when traversing one strand is offset (*k*-1) positions from the strand read from reading in the other direction. This means that after reading *l *nucleotides from the chain in the one direction as *s*, and reading (*k *- 1) nucleotides in the opposite direction as *e*, the full sequence read can be written as *se'*, where *e' *is the complementary strand of *e*.

## Results and Discussion

### Synthetic data

The Illumina sequencing machine currently reports 36 length reads with the ability to report 50 length reads currently in testing. We analyzed data from a single Illumina run from the Michael Smith Genome Sciences Center to produce a model for the generation of vast amounts of synthetic data. The Illumina quality file consists of a vector ⟨*Q*_*A*_, *Q*_*C*_, *Q*_*G*_, *Q*_*T*_⟩, where *Q*_*N *_is the quality score for calling the nucleotide *N*, calculated using the following formula, where *p *is the probability of the nucleotide being *N*:

$$Q=10log_{10}\left(\frac{p}{1-p}\right)$$

The *Q *values are integers in the range [-40, 40], with *Q *= -40 ↔ *p *= 0, *Q *= 0 ↔ *p *= .5 and *Q *= 40 ↔ *p *= 1. To measure the goodness of a base call, we look at the difference between the highest *Q *value and the second highest *Q *value. We want this difference to be significant to consider the call to be valid. For our analysis we chose to consider a difference greater than 10 between the maximum *Q *value and second highest *Q *value to be significant. This corresponds to an underlying probability difference of between .4 and .5.

To adequately generate synthetic data, we are interested in three questions about the Illumina sequence quality: 1) What is the probability that the base call is bad at a particular position (between 1 and 36)? 2) What is the probability that a base call is bad at a particular position, given no bad base calls in a previous position? 3) What is the probability that a base call is bad at a particular position, given some bad base call in a previous position?

**Figure 4:**
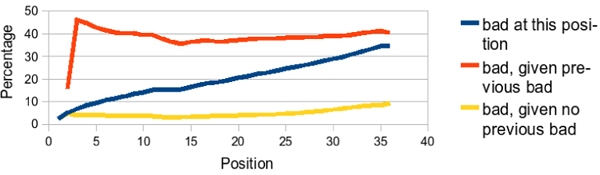
**Error analysis of Illumina data**. Error Analysis of Illumina Data by position. We analyzed the percentage of bad bases (center line), the percentage of bad bases, given some bad base in a previous position (top line), and the percentage of bad bases, given no bad base in a previous position (bottom line).

As can be seen in Fig. 4, the conditional probability that a base is bad if we have previously seen a bad base is high in Illumina data. Conversely, the probability that a base is bad given that all previous bases are good remains low across all positions. From this data, we can infer that once a bad base call is made, whatever condition caused this state remains in effect for the remainder of the base calls, causing the rest of the sequence to be unreliable. At the same time, the sequence before this switchover point is of high quality. For this reason, it seems reasonable to model properly trimmed Illumina sequences as nearly perfect sequences, and we do so by randomly selecting read lengths between 30 and 50.

We generated synthetic data from the genic regions of maize, predicted using FGENESH v.2.6 (using the monocots matrix) on the previously assembled maize genomic islands [1]. We used 61,428 gene structures to generate simulated high coverage transcriptome data. Each gene was sampled at a random coverage between 50× and 1000× using read lengths of 30 to 50 base pairs, resulting in a data set of 925 million reads and 40 billion bases. As discussed in the results section, we assume an adequate preprocessing of the sequences will remove nearly all errors.

### Performance results

We completed performance scalability testing using *p *= 64 to *p *= 1024 and *k *= 30 on a 1024 node Blue Gene/L supercomputer. We timed each stage of the algorithm individually and present the results in Table 1.

**Table 1:** Performance results. Runtime in seconds for the transcriptome data set with approximately 40 billion bases. *p *indicates the number of processors. The last column is the total runtime of all phases, not including file input.

*P*	Read Data	Construct Graph	Compact Graph	Reduce Graph	Total
64	516.47	73.06	81.17	256.8	411.03
128	364.20	40.70	43.17	107.68	191.55
256	189.94	22.63	24.17	59.27	106.07
512	195.04	13.37	15.42	33.23	62.02
1024	168.26	8.08	11.64	20.13	39.85

As can be seen in the table, stages that are not I/O bound achieved a respectable 6:63X speedup when increasing the number of processors from 64 to 512. The reduction in incremental performance towards higher values of *p *is a natural reflection of the problem size becoming smaller per processor. The poor I/O performance is due to the lack of a parallel I/O interconnect on the system tested. As the number of processors increases, the serial interconnect becomes saturated as more processors concurrently read from disk. Disregarding I/O, the assembly of 40 billion bases finished in about 40 seconds using 1024 nodes. Even including serial I/O, the assembly ran in a few minutes.

### Validation and analysis

We analyzed the effect of varying *k *on the resulting compacted graph size and hence the quality of the resulting contigs, as shown in Table 2. As we increase *k*, we see a significant reduction in the number of final contigs produced by our algorithm, from 338,000 for *k *= 20 to 114,000 for *k *= 30. While the relative difference in the number of unique *k*-mers does not change much while varying *k*, the absolute difference in the number of unique *k*-mers is similar to the absolute difference in the output size, which is significant.

**Table 2:** Effect of *k *on graph size. Effect of varying *k *on graph size.

*K*	Unique k-mers	Num Edges	Compacted Edges	Reduced Edges
20	20,537,274	20,658,206	451,718	338,121
25	20,717,553	20,741,818	205,858	149,018
30	20,758,869	20,764,256	154,965	114,028

For *k *= 30 there were approximately two contigs per reference gene. For validation, we used the BLAST tool to align the assembled contigs to the reference. We post-processed the BLAST results to verify that each contig fully aligned to some predicted gene in the reference. Our analysis showed that 92% of the contigs correctly aligned back to the reference. The remaining contigs are mostly the result of over-collapsing edges during graph manipulation. Improving this result is an area of ongoing research.

We also measured how well contigs of length 500 or greater covered the reference sequence. This measure is similar to the *n*50 measure usually used for assessing the quality of a genome assembly, however in our case only a subset of the reference genes will have lengths greater than *n*. We found that approximately 38% of the applicable reference was covered by contigs with length greater than 500. The maximum length contig was 4017. The maximum length contig in the reference was 5704.

## Conclusion

We presented a parallel method for the assembly of unpaired short reads, using a distributed bidirected string graph. In doing so, we address the challenge of effectively manipulating large distributed graphs on parallel computers. We also present a method for making use of variable coverage to resolve conflicts that arise due to repeats. We produce a *de novo *assembly of the *Zea mays *transcriptome, using synthetically generated sequences derived from it. Our method is very fast, producing an assembly of 925 million reads (40 billion nucleotides) in a few minutes. Our final assembly consists of an average of two contigs per predicted gene for this complex plant genome. *De novo *assembly of a genome using short reads will almost certainly require the integration of clone pairs into the proposed method. We are currently working on developing a parallel method for *de novo *genome assembly incorporating clone pair information.

## Competing interests

The authors declare that they have no competing interests.

## Authors' contributions

BJ developed the algorithmic solutions, implemented the software, and drafted the manuscript. PS provided domain expertise, contributed to understanding the problem and the experimental processes, and provided ongoing feedback. SA conceived the problem, critiqued the solution, and assisted in the development and revision of the manuscript.
